# Unraveling lncRNA TRG-AS1: a novel biomarker for poor prognosis of gastric cancer and key to regulating malignant behaviors by targeting miR-873-5p

**DOI:** 10.1186/s41065-025-00459-8

**Published:** 2025-05-29

**Authors:** Miao Hu, Tiesong Zhang, Yuzhen Ma, Huiling Wang, Suping Hou

**Affiliations:** 1 Cancer Treatment Center, Affiliated Hospital of Beihua University, Jilin, 132011 China; 2 Department of Cardiothoracic Surgery, The Fourth Medical Center, Chinese PLA General Hospital, Beijing, 100048 China; 3 Department of Endoscopic Diagnosis and Treatment, Daqing Oil Field General Hospital, Daqing, 163001 China; 4 Department of Clinical Laboratory, Henan Provincial People’s Hospital, People’s Hospital of Zhengzhou University, People’s Hospital of Henan University, Zhengzhou, 450003 China; 5 Pathology, Third Hospital of Hebei Medical University, No. 139, Ziqiang Road, Shijiazhuang, Hebei 050051 China

**Keywords:** TRG-AS, miR-873-5p, Gastric cancer, Prognosis, Cellular processes

## Abstract

**Background and study aims:**

To alleviate patient stress and advance gastric cancer research, this study aims to investigate the potential association between aberrant expression of TRG-AS1 and gastric cancer, and to examine its potential impact on the biological behaviors of gastric cancer cells.

**Materials and methods:**

To find out how TRG-AS1 is expressed in the tissues and cells of gastric cancer, real-time fluorescence quantitative PCR was employed. The connection between TRG-AS1 and pathological features, as well as its prognostic importance, were examined using the Chi-square test and Cox regression analysis. The dual luciferase reporting assay was utilized to confirm the targeting of TRG-AS1 and miR-873-5p. Transwell assay and CCK-8 test were used to identify the roles that TRG-AS1 plays in cell metastasis and proliferation, respectively.

**Results:**

Research has revealed a downregulation of TRG-AS1 in the tissues and cells, which is strongly correlated with the differentiation, TNM stage, lymph node metastasis, depth of invasion, and patient survival rate in gastric cancer. The binding sites exists between miR-873-5p and TRG-AS1, and TRG-AS1 has the ability to negatively control miR-873-5p by acting as a competitive endogenous RNA. When TRG-AS1 was overexpressed, the malignant behavioral activity of gastric cancer cells was significantly decreased; however, the inhibitory effect was reversed when miR-873-5p was overexpressed.

**Conclusions:**

TRG-AS1 is a potential predictor of poor prognosis in gastric cancer patients and targets miR-873-5p to inhibit the progression of cancer.

**Supplementary Information:**

The online version contains supplementary material available at 10.1186/s41065-025-00459-8.

## Introduction

Gastric cancer, as a malignant tumor of the digestive tract, that is highly poses a significant global health burden, representing42% of all incidence rates worldwide with an increasing annual incidence rate [1, 2]. The malignant behavioral characteristicsof gastric cancer cells exert profound impacts on therapeutic outcomes and patient prognosis. Emerging studies have indicated that tumor microenvironment components, epithelial-mesenchymal transition, and genetic regulatory elements play pivotal roles in modulating gastric cancer cell activities. They directly or indirectly activate growth signals in cancer cells or remodel the cellular microenvironment through many pathways, thus promoting tumor growth [3, 4]. For example, transforming growth factor-βreceptor type II down-regulation can inhibit the phosphorylation of SMAD2 and SMAD3, thereby inhibiting the transformation of epithelial morphology to mesenchymal morphology of gastric cancer cells and improving the sensitivity of cancer cells to 5-fluorouracil [5]. Furthermore, loss of TIPRL expression serves as robust indicator for metastatic potential and unfavorable clinical outcomes in gastric cancer, where TIPRL mediates its anti-invasive effects via modulation of the AMPK/mTOR signaling axis [6]. However, current knowledge regarding of the molecular regulatory mechanisms in gastric cancer remains in its nascent stages, highlighting the imperative to identify novel therapeutic targets and elucidate their regulatory networks. These advances will ultimately contribute to refining tumor biology frameworks and optimizing clinical management strategies for this devastating disease.

Long non-coding RNAs (lncRNAs) are a class of transcripts that do not encode any protein. Researchers have discovered that lncRNAs are essential regulators of gene expression in a variety of biological functions and cellular environments, and are involved in transcriptional interference, chromosomal rearrangement, histone modification, splicing and modification of gene sequences, and regulation of protein function. [7, 8]. At present, much evidence has confirmed that lncRNA is related to the pathogenesis of gastric cancer. Some lncRNAs are highly expressed, for instance, gastric cancer cells have elevated levels of the lncRNA DNM3OS, which, upon suppression, dramatically reduces cancer cell proliferation [9]. Kong et al. found that lncRNA PVT1 upregulation was not only related to the TNM stage of patients but also could be used as an independent indicator to predict the survival rate of patients in gastric cancer [10]. On the contrary, certain lncRNA showed a downregulation in gastric cancer, for example, in gastric cancer, lncRNA ELF3-AS1 is down-regulated and contributes to cancer inhibition [11]; LINC01133 is downregulated and inhibits tumor development by negatively regulating miR-106a-3p [12]. However, the identified aberrantly expressed lncRNAs failed to provide strong guidance for intensive treatment of gastric cancer, so the regulatory profile of lncRNAs in gastric cancer remains to be improved.

In this study, 2 differentially expressed lncRNAs were finally screened by bioinformatics analysis: TRG-AS1 and CYP3A51P. CYP3A51P has been subjected to relatively few systematic studies and lacks reference data, leading to its exclusion from further investigation. In contrast, TRG-AS1 has been reported to play an essential regulatory role in a variety of human diseases involving breast cancer [13], colorectal cancer [14], and osteoporosis [15] by participating in diverse network mechanisms. However, in gastric cancer, no reports on its role have been found, so it selected TRG-AS1 as the experimental target to explore its function and mechanism in gastric cancer.

In this study, the expression level of TRG-AS1 in gastric cancer tissues and adjacent normal tissues was detected, and the relationship between TRG-AS1 and the pathological and clinical prognosis of patients was analyzed. The effect of TRG-AS1 on the proliferation and metastasis of gastric cancer cells was analyzed by vitro cell experiments, and the downstream miRNA of TRG-AS1 was further verified by biological analysis and luciferase reporter gene. To provide more theoretical basis for the pathogenesis, therapeutic target selection, and survival prediction of gastric cancer.

## Materials and methods

### Clinical specimens

Paired normal gastric tissue and gastric cancer tissue samples from 133 gastric cancer patients who underwent gastrointestinal surgery at Henan Provincial People’s Hospital from 2016 to 2018. Inclusion criteria: (1) gastric cancer was diagnosed by gastroscopy and pathological tissue biopsy; (2) The Ethics Committee of Henan Provincial People’s Hospital gave its approval for the collecting of all samples, and the subjects voluntarily signed the informed consent for this trial. Exclusion criteria: (1) patients with adjuvant therapy such as radiotherapy and chemotherapy or systemic therapy before surgery; (2) patients with other tumor metastasis or other tumors; (3) patients with severe infection, respiratory insufficiency, coagulation dysfunction, and severe hepatic and renal insufficiency.

## Cell culture and cell transfection

In this experiment, human gastric cancer cell lines AGS, HGC-27 MKN-45, and SNU-16 and human gastric mucosa normal epithelial cells GES-1 (ATCC, USA) were cultured in a carbon dioxide cell incubator at 37℃ and 5%. Cell maintenance culture medium was DMEM, 10% fetal bovine serum (FBS) and 1% penicillin-streptomycin (Gibco, USA) were added. After diluting the transfection sequence and lipofectamine 2000 (Invitrogen, USA) to a specific concentration with serum-free culture medium, the two were put on a 12-well plate. Following mixing, they were cultivated for six hours at 37 °C with 5% CO_2_. Replace it with the complete DMEM medium that contains serum. Cells were collected 36 h after transfection for follow-up experiments.

## Bioinformatics analysis

The datasets about differentially expressed lncRNAs in gastric cancer were screened by the GEO database (https://www.ncbi.nlm.nih.gov/gds). Venn diagrams were plotted using the sieved datasets to obtain the intersecting lncRNAs. The expression profiles of the above lncRNAs in tumor tissues were then searched through the GEPIA (http://gepia.cancerpku.cn/index.html) database.

## qRT-PCR

TRG-AS1 expression was detected by real-time fluorescence quantitative polymerase chain reaction (PCR), and total RNA was extracted using Tizol kit (Invitrogen, USA). RNA was reverse transcribed into complementary DNA using PrmeScriptRT reagent Kit (TaKaRa, Japan). Amplification was performedon a 7500 real-time fluorescent quantitative PCR system (ABI, USA) using the TB Green Premix Ex Taq II PCR Amplification Kit (TaKaRa, Japan). The primer sequences were as Table S1. The PCR cycling conditions were as follows: 95 °C for 30 s, followed by 40 cycles of 95 °C for 5 s and 60 °C for 34 s. GAPDH and U6 were used as internal standards for TRG-AS1 and miR-873-5p, respectively, and the relative expression was calculated using the 2^−ΔΔct^ method.

## Nucleoplasm separation assay

The cytoplasm of the cells was separated from the nucleus using the PARIS Nucleoplasm Separation Kit (Thermo, USA). Then, the levels of lncRNA TRG-AS1 and miR-873-5p were detected in the cytoplasm and nucleus, respectively. u6 and GAPDH were used as internal references of the nucleus and cytoplasm, respectively.

### Cell proliferation assay

For CCK8 assay, the desired cells were taken for serum starvation for 12 h, then inoculated into 96-well plates and continued to be cultured, and CCK8 reagent was added at 24, 48, 72, and 96 h post-culture, respectively, and finally, the optical density was detected at the wavelength of 450 nm for each group of cells.

For plate clone formation experiments, transfected cells were digested, resuspended, counted, and inoculated in 6-well plates. The plates were placed in a suitable environment for incubation, and the medium was changed regularly. After 2 weeks, the plates were fixed with 4% paraformaldehyde and stained with crystal violet. After washing and drying, photographs were taken.

## Transwell assay

T The required cells were starved for 24 h before the migration experiment, after which they were resuspended in serum-free culture medium and added to the upper chamber of the Transwell chamber. The lower chamber was then filled with a medium containing serum. Basement membrane needs to be applied to the top chamber’s bottom for the invasion experiment. Transfer to 37℃, 5% CO_2_ incubator for 48 h. Following PBS cleansing, it was fixed for one hour in anhydrous methanol and stained for two hours in 0.2% crystal violet. The cells that had been moved to the lower chamber were seen and counted using an inverted microscope.

## Dual-luciferase reporter assay

The binding site between TRG-AS1 and miR-873-5p was found by lncRNASNPv3 (http://www.gong_lab.hzau.edu.cn/lncRNASNP3). The wild-type (WT-TRG-AS1) and mutant (MT-TRG-AS1) plasmids were constructed using a pGL3 luciferase vector. Then, according to 2.2 transfection mode, the plasmid was co-transfected into HGC27 and MKN45 with miR-NC, miR-873-5p mimic, and miR-873-5p inhibitor, respectively. After 48 h, luciferase activity was detected.

### Statistical analysis

GraphPad Prism 7.0 and SPSS 23.0 were utilized for data analysis and mapping. Measurement data were expressed as mean ± standard deviation. When the T-test was employed to compare the groups, the independent samples T-test is applied if the data meet the assumptions of normality. The normality is tested by the Shapiro-Wilk test. The homogeneity of variances is tested by Levene’s test. If the assumptions are violated, the Welch’s T-test is used instead. The relationship between TRG-AS1 and clinicopathological features was determined by χ2 test. For the χ2 test, the expected frequency in each cell is checked. It is ensured that the expected frequency is greater than 5. If not, Fisher’s exact test is employed. Prognostic factors and survival curves were analyzed by Cox proportional risk model. Survival curves are plotted by the Kaplan-Meier method. The log-rank test is used to compare the survival curves between different groups. *p* < 0.05 was statistically significant.

## Results

### TRG-AS1 expression and clinical relevance in gastric cancer

The datasets GSE161291, GSE183538 and GSE192468 obtained from the GEO database selection were intersected, and finally 2 differentially expressed lncRNAs were obtained: TRG-AS1, CYP3A51P. Through the GEPIA database, CYP3A51P-related expression profiles were not found, but it was found that TRG-AS1 was remarkably down-regulated (Figure S1) in STAD (stomach adenocarcinoma).

**Fig. 1:**
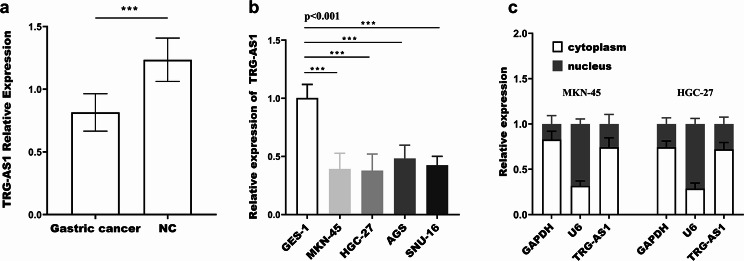
Downregulation and subcellular localization of TRG-AS1 in gastric cancer. (**a**) TRG-AS1 expression expression was significantly downregulated in 133 paired gastric cancer tissues compared with adjacent normal tissues (*p* < 0.001, paired t-test). (**b**) Reduced TRG-AS1 levels were observed in four gastric cancer cell lines (AGS, HGC-27, MKN-45, SNU-16) versus normal GES-1 cells (*p* < 0.001, one-way ANOVA). (**c**) Cytoplasmic predominance of TRG-AS1 was confirmed by nucleoplasm separation assay (*n* = 3 replicates), with U6 and GAPDH as nuclear/cytoplasmic controls

**Fig. 2:**
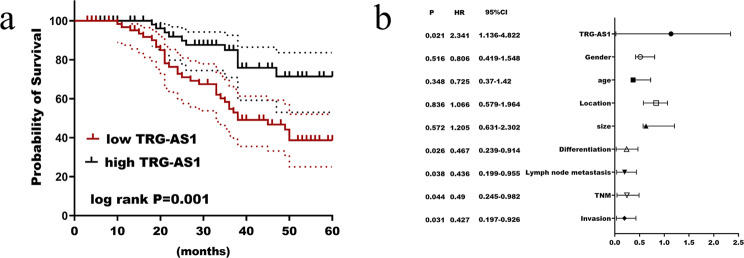
Prognostic significance of TRG-AS1 in gastric cancer. (**a**) Kaplan-Meier survival curves demonstrated significantly shorter overall survival in patients with low TRG-AS1 expression. (**b**) Multivariate cox regression factors affecting prognosis in patients with gastric cancer

qRT-PCR results demonstrated that gastric cancer tissue specimens had substantially lower relative expression of TRG-AS1 than adjacent tissues (Fig. 1a), and it was also significantly lower in the four cancer cells than in GES-1 cells (Fig. 1b). In addition, nucleoplasm separation experiments showed that TRG-AS1 was centrally distributed in the cytoplasm (Fig. 1c). 133 individuals with gastric cancer were divided into two groups based on the average relative expression of TRG-AS1, which was 0.815. Kaplan-Meier survival analysis showed that overall survival was substantially correlated with TRG-AS1 (log rank *p* = 0.001), and low expression of TRG-AS1 predicts poor survival (Fig. 2a). Multivariate Cox regression analysis (Fig. 2b) showed that TRG-AS1 (*p* = 0.021, HR = 2.341), differentiation (*p* = 0.026, HR = 0.467), lymph node metastasis (*p* = 0.038, HR = 0.436), TNM stage (*p* = 0.044, HR = 0.49), depth of invasion (*p* = 0.031, HR = 0.427) can be applied to patients as a prognostic factor. Combined with clinicopathological data (Table 1), it was found that there were marked differences in tumor differentiation (*p* = 0.036), TNM stage (*p* = 0.041), lymph node metastasis (*p* = 0.011) and depth of invasion (*p* = 0.036) between the high and low TRG-AS1 expression groups.

**Table 1:** The association of TRG-AS1 with patients’ clinicopathological features

Variant	Cases (*n* = 133)	TRG-AS1 expression		*P*
		Low (*n* = 67)	high (*n* = 66)	
Gender				0.069
Male	69	40	29	
Female	64	27	37	
Age				0.79
< 45	62	32	30	
≥ 45	71	35	36	
Location				0.261
Proximal	64	29	35	
Middle/Distal	69	38	31	
Tumour size(cm)				0.546
≤ 5	63	30	33	
> 5	70	37	33	
Differentiation				0.036
Well-moderate	85	37	48	
Poor	48	30	18	
Lymph node metastasis				0.011
No	91	39	52	
Yes	42	28	14	
TNM stage				0.041
I-II	94	42	52	
III	39	25	14	
Invasion depth				0.036
T1/T2	85	37	48	
T3	48	30	18	

### TRG-AS1 acts as a CeRNA for miR-873-5p in gastric cancer cells

**Fig. 3:**
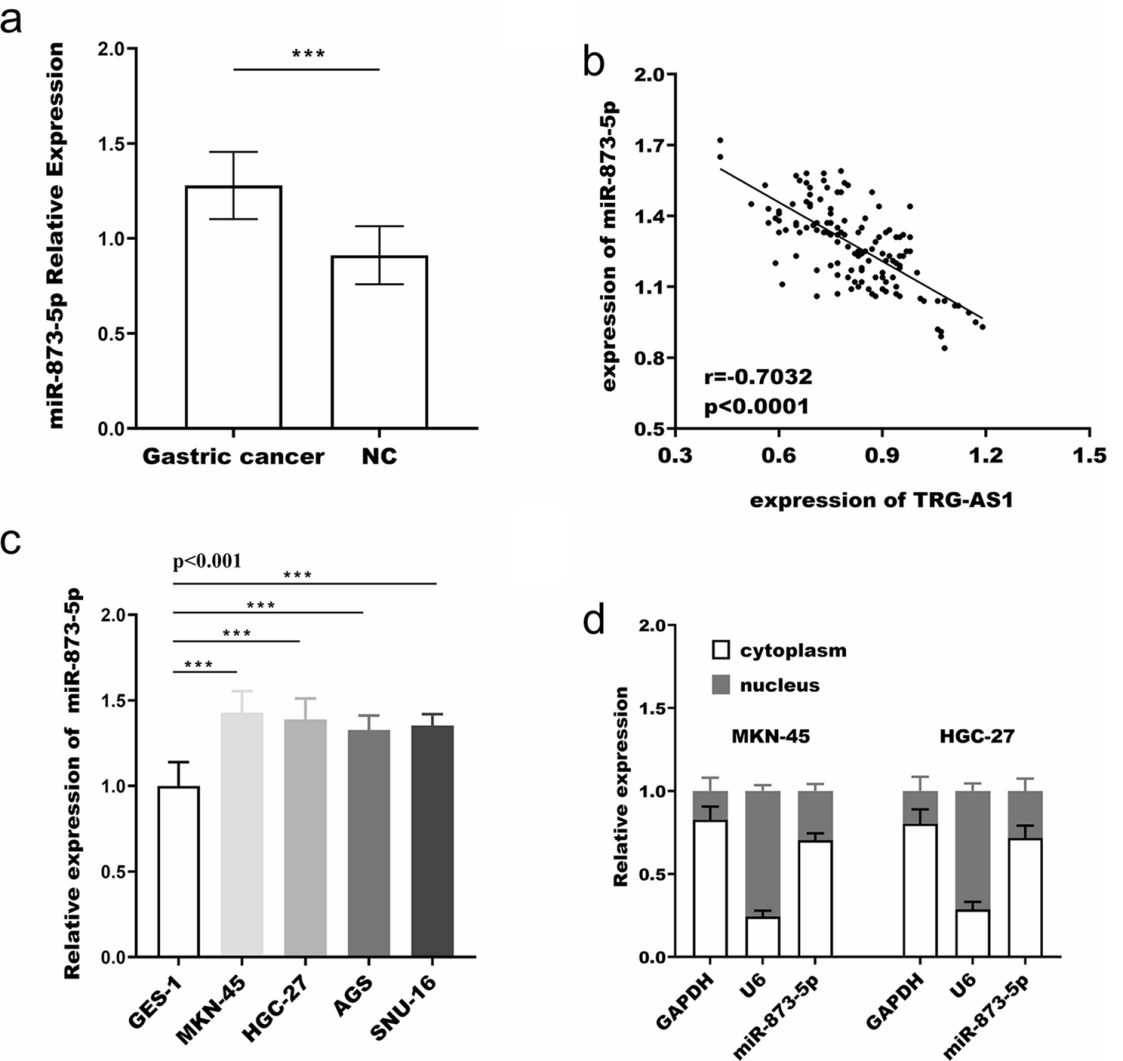
Inverse correlation between TRG-AS1 and miR-873-5p. (**a**) miR-873-5p was upregulated in gastric cancer tissues versus matched normal controls (*p* < 0.001, paired t-test). (**b**) Pearson correlation analysis revealed a negative relationship between TRG-AS1 and miR-873-5p (*r*=-0.703, *p* < 0.0001). (**c**) Elevated miR-873-5p expression was detected in gastric cancer cell lines (*p* < 0.001 vs. GES-1, one-way ANOVA).(**d**) Cytoplasmic localization of miR-873-5p was confirmed

**Fig. 4:**
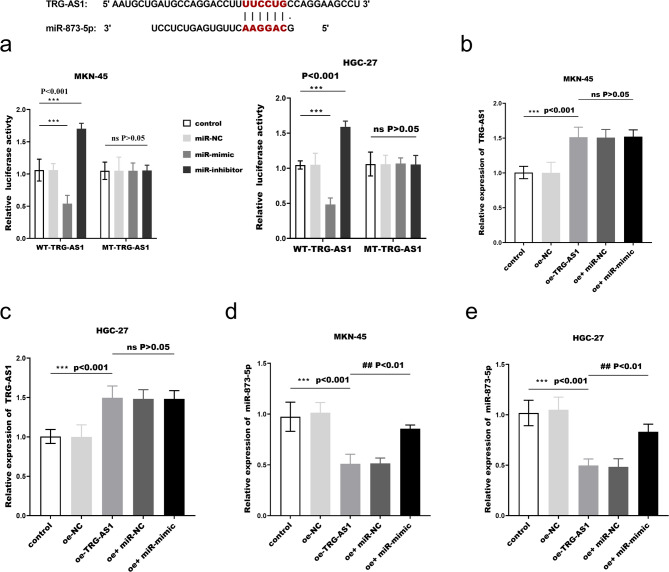
TRG-AS1 directly regulates miR-873-5p. (**a**) Dual-luciferase reporter assay in HGC-27 and MKN-45 cells co-transfected with WT-TRG-AS1 or MT-TRG-AS1 plasmids and miR-873-5p mimics/inhibitors (** *p* < 0.01 vs. control). (**b**)-(**c**) qRT-PCR validation of TRG-AS1 overexpression (oe-TRG-AS1) in transfected cells (** *p* < 0.01 vs. control). (**d**)-(**e**) miR-873-5p expression following TRG-AS1 overexpression (**p < 0.01vs control, ##*p* < 0.01 vs. oe-TRG-AS1). Data analyzed by one-way ANOVA with Tukey’s post hoc test

miR-873-5p expression was upregulated in gastric cancer tissues (Fig. 3a) and was discovered to be inversely linked with the relative expression level of TRG-AS1 (*r*=-0.703, *p* < 0.0001, Fig. 3b). In addition, miR-873-5p expression was upregulated in gastric cancer cells (Fig. 3c) and nucleoplasm separation experiments showed that miR-873-5p was centrally distributed in the cytoplasm (Fig. 3d). The relative luciferase activity of WT-TRG-AS1 was significantly decreased in MKN-45 and HGC-27 cells after co-transfection with miR-873-5p mimic; conversely, activity increased. However, the luciferase activity of MT-TRG-AS1 remained unchanged (Fig. ors of 4a). This suggests that miR-873-5p is a direct target of TRG-AS1. After transfection of the oe-TRG-AS1 fragments, TRG-AS1 expression was upregulated (Fig. 4b-c), which decreased the expression of miR-873-5p (Fig. 4d-e). In summary, the above suggests that TRG-AS1 may function as a ceRNA to control miR-873-5p in gastric cancer.

### TRG-AS1 effects on cell biological behavior

**Fig. 5:**
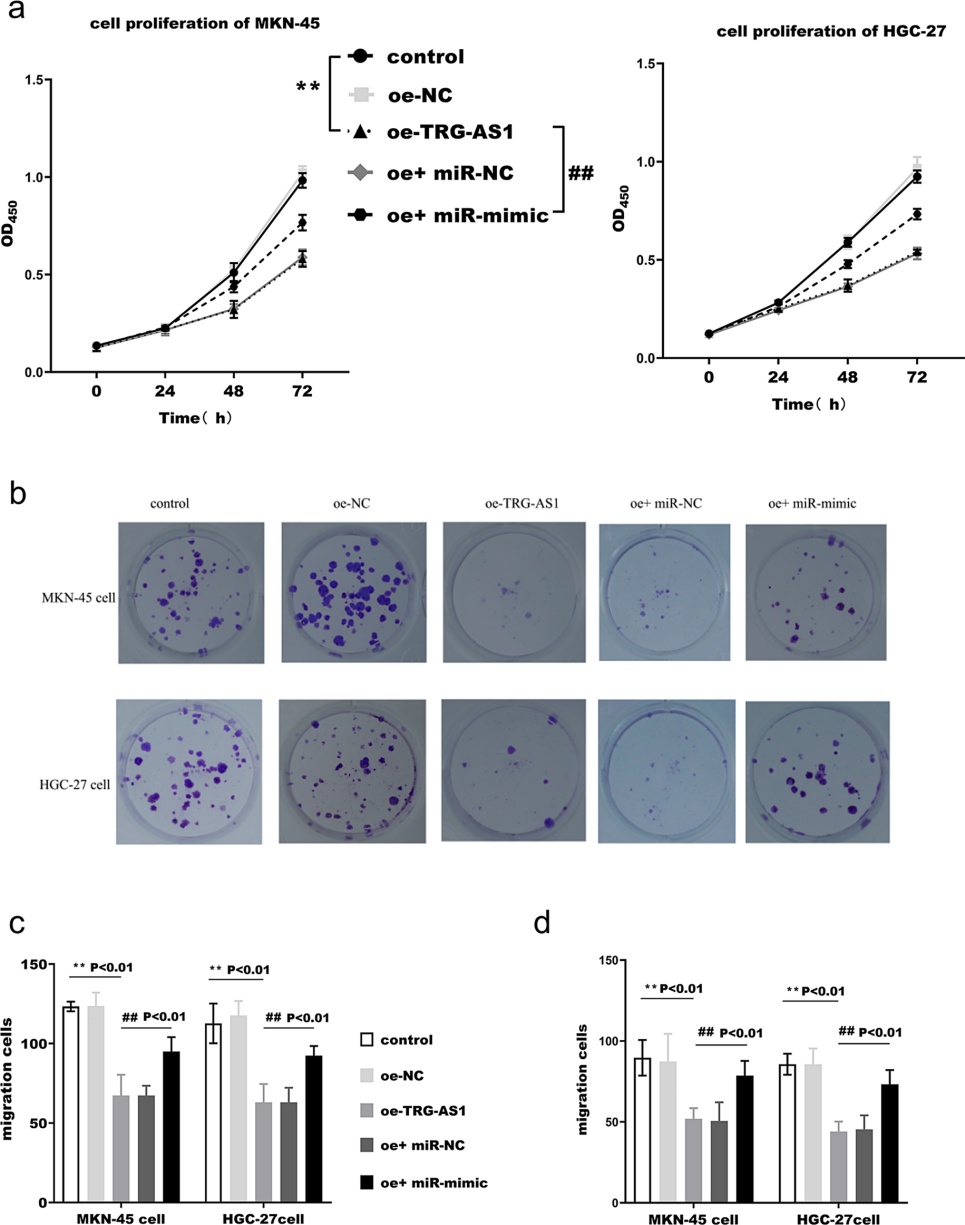
Effects of TRG-AS1 and miR-873-5p on malignant behaviors of cancer cells. (**a**) The proliferative capacity of HGC-27 and MKN-45 cells after TRG-AS1 overexpression and miR-873-5p rescue was assessed by CCK-8 assay. (**b**) Colony formation assay quantified clonogenic capacity after TRG-AS1 overexpression and miR-873-5p restoration. (**c**)-(**d**) Transwell migration and Matrigel invasion assays evaluated metastatic potential under TRG-AS1/miR-873-5p modulation. (*p < 0.01 vs. control; ##*p* < 0.01 vs. oe-TRG-AS1 group, one-way ANOVA)

CCK8 assay indicated that TRG-AS1 overexpression could inhibit the proliferation capacity of MKN-45 and HGC-27 (Fig. 5a). Similarly, plate clone formation assays indicated a dramatic reduction in the number of cells proliferating after TRG-AS1 upregulation (Fig. 5b). Transwell assay indicated that TRG-AS1 overexpression could inhibit their ability to migrate (Fig. 5c) and invade (Fig. 5d). The inhibitory impact of TRG-AS1 was reversed by overexpressing miR-873-5p.

## Discussion

Molecularly targeted therapy is more specific and selective, which greatly reduces the side effects compared to traditional treatment [16, 17]. Several investigations have demonstrated that lncRNAs exhibit differential expression in gastric cancer cells and may find application as tumor markers. The expression of lncRNA LUCAT1 is elevated, and its knockdown can effectively reduce the rate at which gastric cancer cells proliferate and metastasize [18]. Reduced expression of lncRNA-RMRP in gastric cancer tissues is inversely linked with clinical stage and distant metastases [19]. So, the research focuses on mining novel lncRNAs as therapeutic targets and prognostic markers for gastric cancer, providing a strong basis for advancing clinical treatment.

TRG-AS1 was identified as remarkably down-regulated in STAD in the GEPIA database, and the present study similarly found that TRG-AS1 was under-expressed in gastric cancer, and a strong correlation between its expression and tumor development, metastasis, and a poorer prognosis following surgery. He et al., exploring squamous carcinoma of the tongue, found that abnormal expression of TRG-AS1 suggests a poor prognosis and that a warning sign of lymph node metastases could be elevated TRG-AS1 expression [20]. Therefore, it is inferred that lncRNA TRG-AS1 may play a great role in the treatment of gastric cancer and the prognostic judgement of the tumor. The ability of cancer cells to clone malignantly and early distant metastasis are the most difficult hurdles to overcome in cancer treatment. Consequently, knowing how TRG-AS1 affects the proliferation, migration, and invasion of cancer cells will be crucial information for enhancing treatment and prognosis. According to our research, overexpression of TRG-AS1 can reduce the malignant behavioral activities of gastric cancer cells, including proliferation, migration, and invasion. In glioblastoma, TRG-AS1 has also been shown to control cell growth and proliferation in addition to being a biomarker of a bad prognosis [21]. Similarly, TRG-AS1 is abnormally expressed in liver cancer tissues, and TRG-AS1 silencing can significantly inhibit the proliferation and growth of cancer cells, as well as metastasis [22]. It can be inferred from this that TRG-AS1 is associated with the regulation of gastric cancer cell proliferation and metastasis.

According to studies, lncRNAs have a variety of roles in the formation of tumors, which can adsorb miRNAs as sponges in the form of ceRNAs thereby regulating the expression and function of relevant genes, etc., and influencing signal transduction pathways to regulate cellular metabolism [23, 24]. TRG-AS1 was reported by Zhu et al. to sponge miR-877-5p, hence inhibiting cancer bone metastasis [25]. TRG-AS1/miR-224-5p controls proliferative and invasive activity, according to Zhang et al.’s findings in lung cancer [26]. Through investigating the downstream miRNA of TRG-AS1, we discovered that in gastric cancer, TRG-AS1 may function as a ceRNA to regulate miR-873-5p and that overexpressing miR-873-5p could reverse the inhibitory effect of TRG-AS1 overexpression on cancer cells. In colorectal cancer, miR-873-5p prevents metastasis of cancer cells by targeting ZEB1, which is involved in epithelial-mesenchymal transition [27]. miR-873-5p regulates the invasive process of papillary thyroid carcinoma cells by targeting CXCL16, which has a structural domain of a chemokine [28]. miR-873-5p inhibits the development of colorectal cancer by regulating TUSC3/AKT signaling [29]. It was hypothesized that TRG-AS1 probably plays a pivotal regulatory role in the progression of gastric cancer by negatively regulating miR-873-5p.

However, due to time constraints, the downstream target ge nes of miR-873-5p were not further predicted and analyzed in this study. Chen et al. found that miR-873-5p regulates the malignant behavioural activities of tumour cells and affects chemotherapy resistance by targeting THUMPD1 in gastric cancer [30]. The regulatory relationship between TRG-AS1/miR-873-5p and THUMPD1 deserves further in-depth investigation. In the follow-up study, the specific biological roles of TRG-AS1/miR-873-5p in gastric cancer will be clarified by further exploring the target genes of miR-873-5p and the affected signaling pathways in gastric cancer cells. In addition, more experiments, such as EDU, FISH, and animal experiments, will be conducted to increase the scientific validity and reliability of this study, so as to lay a more sufficient experimental foundation for the clinical application of TRG-AS1/miR-873-5p in gastric cancer patients.

In conclusion, it reveals the critical role of TRG-AS1 in gastric cancer, deepens the understanding of the pathogenesis of gastric cancer, and promotes the clinical application of TRG-AS1.

## Electronic supplementary material

Below is the link to the electronic supplementary material.

Supplementary Material 1: Figure S1 (a) Venn diagram of differentially expressed lncRNAs from GEO datasets (GSE161291, GSE183538, GSE192468). (b) TRG-AS1 expression across multiple cancer types in the GEPIA database

Supplementary Material 2

## Data Availability

The datasets used and/or analysed during the current study are available from the corresponding author on reasonable request.
