# Supplementary material for: Unraveling lncRNA TRG-AS1: a novel biomarker for poor prognosis of gastric cancer and key to regulating malignant behaviors by targeting miR-873-5p
Source: Hereditas. 2025 May 29;162:88. doi: 10.1186/s41065-025-00459-8 (PMC12121179; doi:10.1186/s41065-025-00459-8)
Supplement: Supplementary file 2 — Supplementary Material 2 [file 41065_2025_459_MOESM2_ESM.docx]

Table S1 Primer sequences

| Primer name | | Primer sequence |
| --- | --- | --- |
| TRG-AS1 | forward | 5'-GGAGTCTGCTCTAAGAGCTG-3' |
|  | reverse | 5'-CAGAGCAAAGATGCTCTGC-3'' |
| GAPDH | forward | 5'-CGACCACTTTGTCAAGCTCA-3' |
|  | reverse | 5'- ACTGAGTGTGGCAGGGACTC-3' |
| miR-873-5p | forward | 5'-CGGCGGGCAGGAACTTGTGAGT-3' |
|  | reverse | 5'-CTGGTGTCGTGGAGTCGGCAATTC-3' |
| U6 | forward | 5'-GCTTCGGCAGCACATATACTAA-3' |
|  | reverse | 5'-AACGCTTCACGAATTTGCGT-3' |
